# Harnessing cutting-edge techniques to identify novel gene expression signatures in acute myeloid leukemia patients

**DOI:** 10.3389/fbinf.2026.1805826

**Published:** 2026-06-08

**Authors:** Adriana Blanda, Rebecca Manitto, Sara Pizzamiglio, Sabina Sangaletti, Paolo Verderio

**Affiliations:** 1 Unit of Bioinformatics and Biostatistics, Fondazione IRCCS Istituto Nazionale Dei Tumori, Milan, Italy; 2 Unit of Molecular Immunology, Fondazione IRCCS Istituto Nazionale Dei Tumori, Milan, Italy

**Keywords:** acute myeloid leukemia, cross-validation, machine learning, performance metrics, survival analysis

## Abstract

**Introduction:**

Acute Myeloid Leukemia (AML) is a heterogeneous hematological malignancy with poor prognosis, despite therapeutic advances. Gene expression analysis has emerged as a powerful tool for identifying novel biological markers that can aid in predicting patient outcomes. This study is aimed to identify prognostic gene expression signatures from RNA-seq data of 457 AML patients.

**Methods:**

Filtering methods were applied to reduce the set of genes, resulting in a subset of 685. Lasso-Cox, Bayesian Model Averaging (BMA), Random Survival Forest (RSF), and a Cox-time Neural Network (CtNN) were implemented to select the most promising prognostic genes. Explainable tools such as SHapley Additive exPlanations (SHAP) and Local Interpretable Model-agnostic Explanations (LIME) were used to gain a deeper understanding of neural network outputs. An enrichment analysis was performed to identify enriched biological pathways.

**Results:**

SHAP and LIME interpretations of the CtNN output identified gene sets that differed from those selected by the other algorithms. According to the two-validation metrics the SHAP-derived signature demonstrated superior performance in the testing set. (C-index[95%CI]: 0.639[0.581–0.696] and Integrated Brier Score [95%CI]: 0.172[0.161–0.183]).

**Discussion:**

Enrichment analysis revealed structural and developmental pathways, emphasizing the role of microtubule dynamics and ciliary-related signaling in the bone marrow microenvironment.

## 1 Introduction

Acute Myeloid Leukemia (AML) is a rare and aggressive tumor of the hematopoietic tissue. The disease is characterized by an abnormal proliferation of blood cells in the bone marrow. In the western world, leukemia is one of the most common cancers (Isidori et al., 2021). Despite intensive chemotherapy followed by allogeneic stem cell transplantation and the recent approval of new targeted agents, the overall outcome of AML is still unsatisfactory, with an estimated 5-year overall survival (OS) of approximately 30% (Suguna et al., 2018). Despite advances in our understanding of the disease and its treatment, an unmet medical need still remains. It is believed that AML is caused by genetic predisposition, but it is unclear how this exactly influences the dynamics of disease. In recent years, the use of genomic has become an increasingly powerful tool for determining the management strategies, assessing prognosis, and predicting patient outcome in AML (Padmakumar et al., 2021; Gregory et al., 2009). This approach has greatly facilitated the discovery of new biological markers that are relevant in understanding the disease and its progression. This study is aimed to compare novel gene expression signatures in AML patients using different methodological approaches and to identify which best predict patient survival outcome.

## 2 Data and methods

The cohort was download from the GSE6891 dataset, retrieved from the Gene Expression Omnibus (GEO). It comprises transcriptomic data from two independent and representative cohorts of *de novo* AML patients (≤60 years), totaling 461 individuals. Gene expression profiling was performed using the Affymetrix Human Genome U133Plus2.0 GeneChips (Affymetrix, Santa Clara, CA, United States). The datasets downloaded from GEO were already preprocessed and normalized. The patient cohort with available gene expression profile (n = 457) had a median age of 43 years [IQR: 33–53]. Over a median follow-up period of 205 months [IQR: 45–272], a total of 290 deaths were observed. The dataset was randomly split to create a training (70%, n = 321) and a testing dataset (30%, n = 136). In the training set, a univariate Cox proportional hazards model was trained on each gene. To reduce the number of candidate genes and to perform a preliminary selection, p-value adjustment techniques like False Discovery Rate (FDR, threshold = 0.05) (Storey, 2011) and Hazard Ratio (HR) filter (HR < 0.5 or HR > 2) were applied.

Afterwards, in order to identify gene signatures, starting from the same gene set, a feature selection through algorithms - Lasso-Cox, Bayesian Model Averaging (BMA), Random Survival Forest (RSF) and Non-Proportional Cox-time Neural Network (CtNN) - was carried out in the training set. Based on the gene list selected by each algorithms a specific signature was developed by implementing a multivariate Cox regression model within the training set. The estimated coefficients for each gene were used to generate prognostic signature for each algorithm. Following the training phase, each signature was evaluated in the testing set. The signatures were calculated by applying the fixed coefficients derived from the multivariate Cox models established in the training set.

The performance of the identified signatures was assessed in the testing set and compared according to the following evaluation criteria:

1. Number of features included in the model to ensure parsimony.
2. Harrell’s Concordance index (C-index) (Harrell et al., 1982) to assess the discriminatory capability.
3. Integrated Brier Score (IBS) (Park et al., 2021) to evaluate overall prediction error and calibration over the study period.

Briefly, below some details on the employed methods.

### 2.1 Lasso-cox

Lasso- Cox (Least Absolute Shrinkage and Selection Operator) (Tibshirani, 1997) is a Cox proportional hazards regression model in which L1 regularization is applied in the context of survival data. The algorithm performs an automatic feature selection by shrinking the coefficients of less important variables towards zero, effectively excluding them from the model. The optimal tuning parameter for the Lasso penalty is selected via five-fold cross-validation, balancing model fit and sparsity. In R, the *glmnet* package (Friedman et al., 2010) was used.

### 2.2 Bayesian model averaging

Bayesian Model Averaging (Hoeting et al., 1999) is an iterative algorithm that, in order to select a promising gene subset, combines the effectiveness of *K* multiple models by taking the weighted average of their posterior distributions. Given a total of J covariates of interest, the posterior probability of inclusion for a specific covariate *X*_*j*_(*j* = 1*,* . . . *, J*) is calculated by summing the posterior probabilities of all models that include *X*_*j*_.

$$P\;\left(Xj|D\right)=\sum_{Mk:Xj\in Mk}P\;\left(M_{k}|D\right),$$

where *M*_*k*_(*k* = 1*,* . . . *, K*) is the *k*_th_ model and *D* the observed data. To efficiently explore the high-probability regions of the model space (2^*J*^ possible subsets), BMA relies on the stochastic search algorithm. Metropolis-Hastings based on Markov Chain Monte Carlo method. Therefore, in order to predict survival outcomes, genes with higher posterior inclusion probabilities are selected. Variable selection was assessed using 5-fold cross-validation, implemented with the *caret* package (Kuhn, 2008) in R.

In R, the *iterativeBMAsurv* package (Annest et al., 2009) was used, which provides specialized algorithms for iterative BMA in the survival analysis field.

### 2.3 Random survival forest

Survival Random Forest (Ishwaran et al., 2008) is a non-parametric ensemble method useful for feature selection. It extends the traditional Random Forest algorithm based on Decision Trees to handle censored survival data. The algorithm is built using the set of specific tuning parameters - mtry, ntree, nodesize - to optimize its performance. Feature importance in SRF is assessed using the function Variable Importance (VIMP). Variables with higher VIMP scores are considered more important to predict survival. Cross-validation was inherently supported through the algorithm’s built-in Out-of-Bag (OOB) estimation. In R, the *randomForestSRC* package (Ishwaran et al., 2008) was used.

### 2.4 Non-proportional Cox-time neural network

The Non-Proportional Cox-Time (CtNN) method as described by Kvamme (Kvamme et al., 2019) is a Neural Network-based approach for time-to-event predictions. The CtNN architecture - including three hidden layers, the ReLU (Rectified Linear Unit) activation function, batch normalization and dropout regularization - is designed to accommodate complex non-linear interactions and potential violations of the proportional hazard assumption, which are commonly observed in survival data. Dropout acts as a form of internal stochastic regularization and can be interpreted as an implicit model averaging strategy, helping to reduce overfitting similarly to internal cross-validation mechanisms (Srivastava et al., 2014). It also allows time-varying hazard ratios, thereby generalizing the Cox model by relaxing its inherent assumption of time-invariant covariate effects. The CtNN maps the input features to a set of time-dependent risk scores, which are then used within a modified Cox loss function. This approach can improve prediction accuracy by learning flexible and non-linear transformations of the input features, especially in situations where the proportional hazards assumption is violated. In Python the package *pycox* (Kvamme, 2018) was used.

### 2.5 SHAP and LIME

Due to the intrinsic black-box nature of neural networks, to improve a clinical interpretability, model-agnostic interpretability techniques such as SHAP (SHapley Additive exPlanations) (Marcılio and Eler, 2020) and LIME (Local Interpretable Model-agnostic Explanations) (Garreau and Luxburg, 2020) were adopted. LIME and SHAP were used to visualize and explain the predictions from the CtNN model. In particular, SHAP values provide the contribution of each feature to the difference between the actual prediction and the mean prediction across all instances. Conversely, LIME provides gene influence specific to individual patients. For a given patient, LIME generates perturbed instances of the input data and evaluates the corresponding changes in the model’s output. It then learns a simple, interpretable model around that local neighborhood to explain the individual prediction. In Python the packages *shap* (Lundberg, 2016) and *lime* (Ribeiro, 2016) were used.

### 2.6 Trasparency e explanaibility

Algorithms descripted above can be categorized into two groups based on their nature: transparency and explainability. While methods such as Lasso-Cox and BMA rely on linear coefficients, their intrinsic transparency stems from their capacity for automated feature selection, Random Survival Forest and CtNN operate as complex models where the relationship between features and survival is not immediately apparent. For the Random Survival Forest, explainability is achieved through intrinsic metrics such as Variable Importance (VIMP), which quantifies the contribution of each feature to the model’s predictive accuracy via permutation. For CtNN, instead, explainability tools like SHAP and LIME are necessary to provide *post hoc* interpretations by estimating each feature’s contribution to the model’s decisions (Salih et al., 2025; Roshinta and Gábor, 2024; Wu et al., 2024). While SHAP offers theoretically grounded and consistent explanations based on game theory, LIME provides faster but sometimes less stable local approximations of feature importance (Roshinta and Gábor, 2024; Wu et al., 2024).

### 2.7 Enrichment analysis

To explore the biological functions and potential pathways, gene ontology (GO) (Ashburner et al., 2000) pathway enrichment analysis were performed using the *clusterProfiler* package (Yu et al., 2012) in R. Functional classifications with a false discovery rate (FDR) less than 0.05 were considered significant.

## 3 Results

**FIGURE 1:**
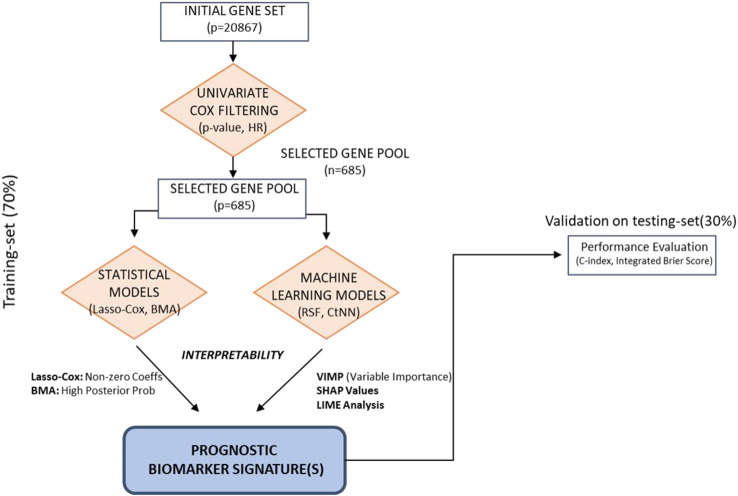
Workflow of analysis.

The comprehensive study workflow is illustrated in Figure 1. The initial analysis was conducted on the training set comprising 20,867 genes and 321 patients. Following the preliminary selection procedure, a subset of 685 genes (designated as the “gene pool”) was identified for further investigation. To explore the biological implications of these findings, the gene pool was stratified based on Hazard Ratios (HR) derived from univariate Cox regression models. Functional enrichment analysis was performed separately for risk genes (defined by an HR greater 2) and protective genes (defined by an HR lower then 0.5).

**FIGURE 2:**
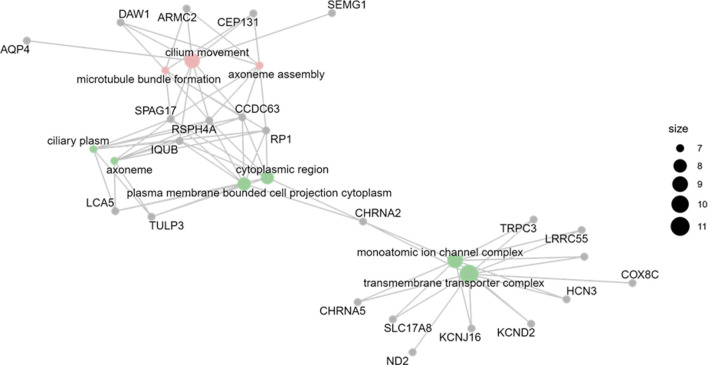
Network plot showing the functional enrichment results for genes with HR *>* 2. Enriched Biological Process (BP) terms are pink nodes, Cellular Component (CC) terms are green. Node size reflects the number of associated genes, and edge thickness reflects the strength of the gene–term association.

**FIGURE 3:**
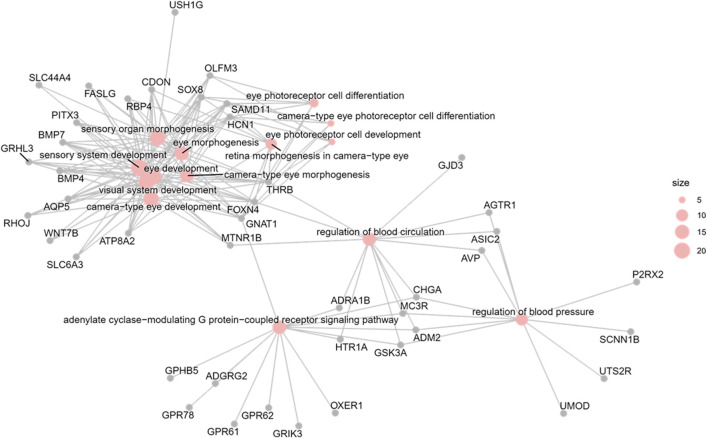
Network plot illustrating the functional enrichment of genes with HR *<* 0.5. Nodes representing enriched Biological Process (BP) terms are pink.

Among the 216 risk genes, 185 were successfully mapped to their respective Entrez Gene IDs using the *org.Hs.e.g.db* human genome annotation database. The enrichment analysis identified three significantly enriched Biological Process (BP) terms and six significantly enriched Cellular Component (CC) terms. These findings are summarized in Figure 2. Of the 469 protective genes, 408 were successfully mapped using the same database. The analysis revealed 14 significantly enriched BP terms. No significant enrichment within other Gene Ontology (GO) categories (Figure 3). Starting from the pre-selected gene pool, various algorithms, ranging from traditional statistical models to complex “black-box” machine learning techniques, were applied to the training set.

**FIGURE 4:**
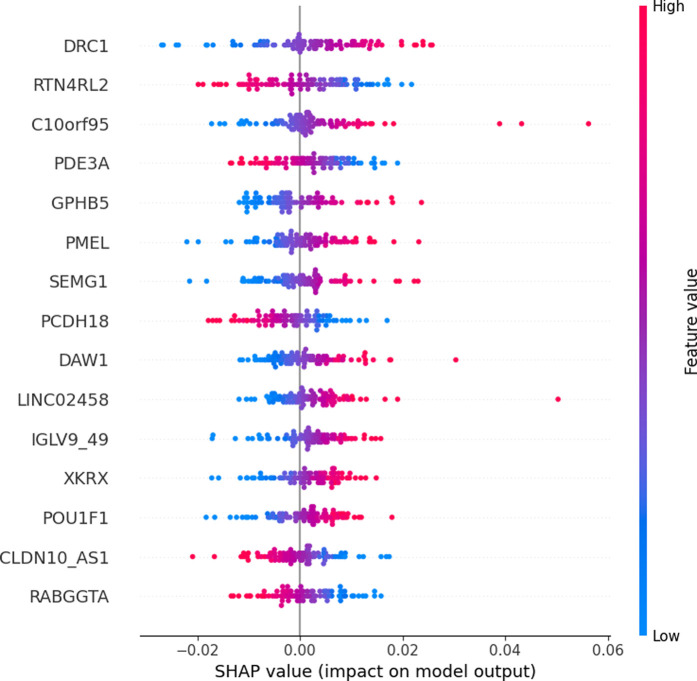
SHAP summary plot. Y-axis: genes ranked by impact; X-axis: SHAP value (positive = increased risk, negative = decreased risk).

**FIGURE 5:**
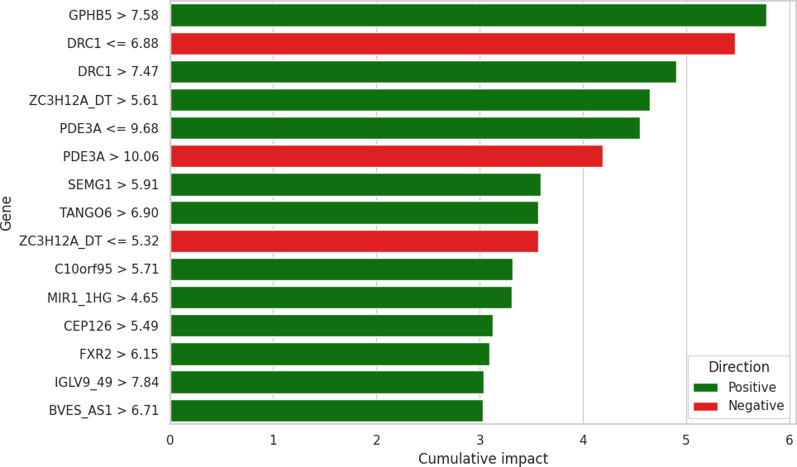
LIME impact plot. Y-axis: genes with their respective threshold; X-axis: cumulative impact of the gene.

Regarding the statistical models, Lasso-Cox regression and BMA selected 3 and 9 genes, respectively. For the “black-box” models, RSF and CtNN, feature selection was performed based on importance metrics by taking the top ten genes. Specifically, RSF features were selected using the VIMP and CtNN features were identified using SHAP and LIME. Detailed lists of the genes selected by each algorithm are provided in Supplementary Table 1. Notably, the intersection among the explainability algorithms yielded a set of five genes: GPHB5, DRC1, PDE3A, SEMG1, and C10orf95. In Figures 4, 5 are reported the top-ranked genes with the most substantial impact on survival, as identified by SHAP and LIME respectively.

Based on the gene lists reported in Supplementary Table 1, a specific signature was developed for each algorithm. As reported in Supplementary Table 1, within the training set, the model performance in terms of C-index ranged from 0.71 to 0.58, with the BMA method achieving the highest discriminative value.

Following the training phase, each signature was evaluated in the testing set. As summarized in Table 1, only two of the identified gene signatures maintained their predictive validity in the testing set: the 3-gene signature derived via Lasso-Cox and the 10-gene signature identified through SHAP analysis. Considering both the C-index and IBS metrics, the SHAP-derived signature demonstrated superior performance. This optimal signature included the following ten genes: DRC1, RTN4RL2, C10orf95, PDE3A, GPHB5, PMEL, SEMG1, PCDH18, DAW1 and LINC02458. Finally, the biological pathways identified through the enrichment analysis of the genes included in these two validated signatures are summarized in Table 2. These results provide a functional overview of the biological processes captured by both the transparency and explainability frameworks.

**TABLE 1:** Performance of gene selection.

Testing set	Trasparency Algorithms		Explainability Algorithms			
	1.Lasso-cox	2.BMA	3.VIMP	4.SHAP	5.LIME	6.SHAP Ո LIME
C-index [95%CI]	0.585 [0.522–0.648]	0.541 [0.474–0.607]<	0.562 [0.497–0.627]	0.639 [0.581–0.696]	0.559 [0.496–0.622]	0.561 [0.495–0.628]
Integrated brier score [95%CI]	0.181 [0.169–0.190]	0.191 [0.180–0.200]	0.183 [0.172–0.194]	0.172 [0.161–0.183]	0.189 [0.179–0.198]	0.187 [0.177–0.198]

**TABLE 2:** Functional categorization and assignment to macro-categories by algorithms.

Transparency algorithms	Explainability algorithms
1. Bile acid and cholesterol metabolism	1. Ciliary function and movement
2. Steroid and lipid metabolism	2. Structural organization and microtubules
3. Response to chemical compounds and vitamins	3. Development and body symmetry
4. Hormone processing and signaling	4. Reproduction and germ cell development
5. RNA modification and nucleoside synthesis	
6. Oocyte development	

## 4 Discussion

This study is aimed to identify prognostic gene expression signatures in AML patients. After a preliminary selection step, various algorithms, ranging from traditional statistical models to complex “black-box” machine learning techniques, were applied to select the most promising prognostic signature. Explainable AI tools such as SHAP and LIME were used to gain a deeper understanding of neural network outputs, highlighting gene-level contributions to survival risk. As a final result, only two of the identified gene signatures were validated in the testing set: the 3-gene signature derived via Lasso-Cox and the 10-gene signature identified through SHAP technique. The genes identified by transparency algorithms highlighted metabolic rewiring, with a specific focus on lipid homeostasis and RNA processing—hallmarks of leukaemic cell metabolic adaptation. In contrast, explainability algorithms identified structural and developmental pathways, emphasising the role of microtubule dynamics and ciliary-related signalling in the bone marrow microenvironment (Zheng et al., 2025). The variety of biological pathways found in the genes selected by each algorithm reflects their underlying computational mechanisms. According to the two considered validation metrics the SHAP-derived signature demonstrated superior performance in the testing set. While traditional methods often rely on linear assumptions, the SHAP-based interpretation of the neural network identified a set of genes that captures complex biological pathways. The genes included in the SHAP-derived signature are DRC1 (Dynein Regulatory Complex Subunit 1), RTN4RL2(Reticulon 4 Receptor Like 2), C10orf95(Chromosome 10 Open Reading Frame 95), PDE3A (Phosphodiesterase 3A), GPHB5 (Glycoprotein Hormone Subunit Beta 5), PMEL (Premelanosome Protein), SEMG1 (Semenogelin 1), PCDH18 (Protocadherin 18), DAW1 (Dynein Assembly Factor with WDR Repeat Domains 1), LINC02458 (Long Intergenic Non-Protein Coding RNA 2458). These gene do not seem to cluster around one dominant pathway. Instead, the most convincing signal is a modest but consistent enrichment for processes related to motile cilia and axonemal structure, largely driven by DRC1 and DAW1 (Wirschell et al., 2013). The remaining genes appear to reflect separate biological themes, including melanosome/pigmentation biology (PMEL), neural-related adhesion pathways (RTN4RL2 and PCDH18), male reproductive functions (SEMG1), and endocrine-related signaling (GPHB5). PDE3A is known for encoding a phosphodiesterase involved in cyclic nucleotide signaling, which can influence cell proliferation and apoptosis, processes relevant to cancer biology (Savai et al., 2010). Interestingly, LINC02458 is a long noncoding RNA (lncRNA), and lncRNAs have been increasingly recognized as important regulators in acute myeloid leukemia (AML) by modulating gene expression related to differentiation, proliferation, and apoptosis of leukemic cell. (Ng et al., 2019). Altogether, PDE3A and LINC02458 could potentially influence AML through regulatory or signaling mechanisms that warrant further investigation. The lack of recurrent mutations or functional studies connecting most of these genes to AML suggests they are not primary drivers but might be explored as part of broader molecular networks affecting leukemogenesis. In summary, PDE3A and LINC02458 have plausible roles related to AML biology through signaling and gene regulation respectively, while the other listed genes currently lack well-established associations with AML in large-scale genomic studies (Ley et al., 2013) but could be considered for exploratory studies on their indirect contributions. To further evaluate the clinical relevance of our findings, we compared the performance of our 10-gene signature with a recently published prognostic signature for AML patients based on neutrophil extracellular trap (NET)-related genes (Wang et al., 2024). When we applied the Wang et al. signature to our dataset, it showed a lack of predictive power with a C-index of 0.529 [95% CI: 0.488–0.570] in the training set and 0.516 [95% CI: 0.452–0.580] in the testing set, giving a C-index of 0.524 [95% CI: 0.490–0.558] in the whole cohort. Notably, as the lower bounds of the 95% confidence intervals across all sets were consistently below or near the 0.5 threshold, the performance of the Wang et al. signature was statistically non-significant in our cohort.

In conclusion, although several of the identified genes lack well-established associations with AML, their involvement in diverse biological processes suggests that they may play a role in broader regulatory networks rather than being primary disease drivers. The limited overlap with previously published signatures further highlights the heterogeneity of AML and the difficulty of deriving universally robust prognostic markers. Overall, this study highlights the usefulness of explainability tools for discovering biomarkers in AML, and identifies a gene signature that require further validation in independent cohorts and functional studies.

## 5 Conclusion

This study investigated different approaches to identified prognostic gene signatures in AML patients. Specifically, Lasso-Cox algorithm identified the most parsimonious signature with 3 genes. The signature of 9 genes identified by BMA was those with the highest performance in the training set. Among the 3 signatures obtained by the explainability algorithms, all composed of the top 10 genes selected according to the appropriate metric, only the signature obtained by SHAPE was validated in the testing set. Interestingly, despite starting from the same set of genes, the algorithms highlighted different subsets of relevant genes. The two algorithms with the greatest overlap in terms of selected genes are SHAP and LIME. According to the two considered validation metrics the SHAP-derived signature demonstrated superior performance in the testing set. This may be attributed to the ability of explainability technique to uncover non-linear patterns and complex biological pathways.

## Data Availability

The datasets presented in this study can be found in online repositories. The names of the repository/repositories and accession number(s) can be found below: https://www.ncbi.nlm.nih.gov/geo/, GSE6891.
