# Supplementary material for: Harnessing cutting-edge techniques to identify novel gene expression signatures in acute myeloid leukemia patients
Source: Front Bioinform. 2026 Jun 8;6:1805826. doi: 10.3389/fbinf.2026.1805826 (PMC13284140; doi:10.3389/fbinf.2026.1805826)
Supplement: Supplementary file 1 [file Table1.pdf]

Suppelementary table 1. List of gene signatures selected by each algorithm.

Training set	*Trasparency Algorithm*		*Explainability Algorithm*			
**#**	**1.Lasso-Cox**	**2.BMA**	**3.VIMP**	**4.SHAP**	**5.LIME**	**6.SHAP ∩ LIME**
1	CDCP1	TMEM221	C8G	DRC1	GPHB5	GPHB5
2	CA13	TEX36_AS1	ZNF560	RTN4RL2	DRC1	DRC1
3	MALRD1	RPUSD1	C12ORF50	C10orf95	ZC3H12A_DT	PDE3A
4		LINC02361	TEX36_AS1	PDE3A	PDE3A	SEMG1
5		ZGLP1	LYPD5	GPHB5	SEMG1	C10orf95
6		CDCP1	CA13	PMEL	TANGO6	
7		LINC02603	TEDC2_AS1	SEMG1	C10orf95	
8		TEDC2_AS1	MALRD1	PCDH18	MIR1_1HG	
9		BCHE	EP300_AS1	DAW1	CEP126	
10			USF2	LINC02458	FXR2	
**C-Index [95%Confidence Interval]**	0.612 [0.581-0.661]	0.709 [0.675-0.744]	0.669 [0.632-0.706]	0.597 [0.555-0.638]	0.598 [0.556-0.639]	0.576 [0.537-0.616]
